# Supplementary material for: Prevention and Therapy of Metastatic HER-2+ Mammary Carcinoma with a Human Candidate HER-2 Virus-like Particle Vaccine
Source: Biomedicines. 2022 Oct 20;10(10):2654. doi: 10.3390/biomedicines10102654 (PMC9599132; doi:10.3390/biomedicines10102654)
Supplement: Supplementary file 1 [file biomedicines-10-02654-s001.zip › biomedicines-1919775-supplementary.pdf]

Supplementary Figure S1

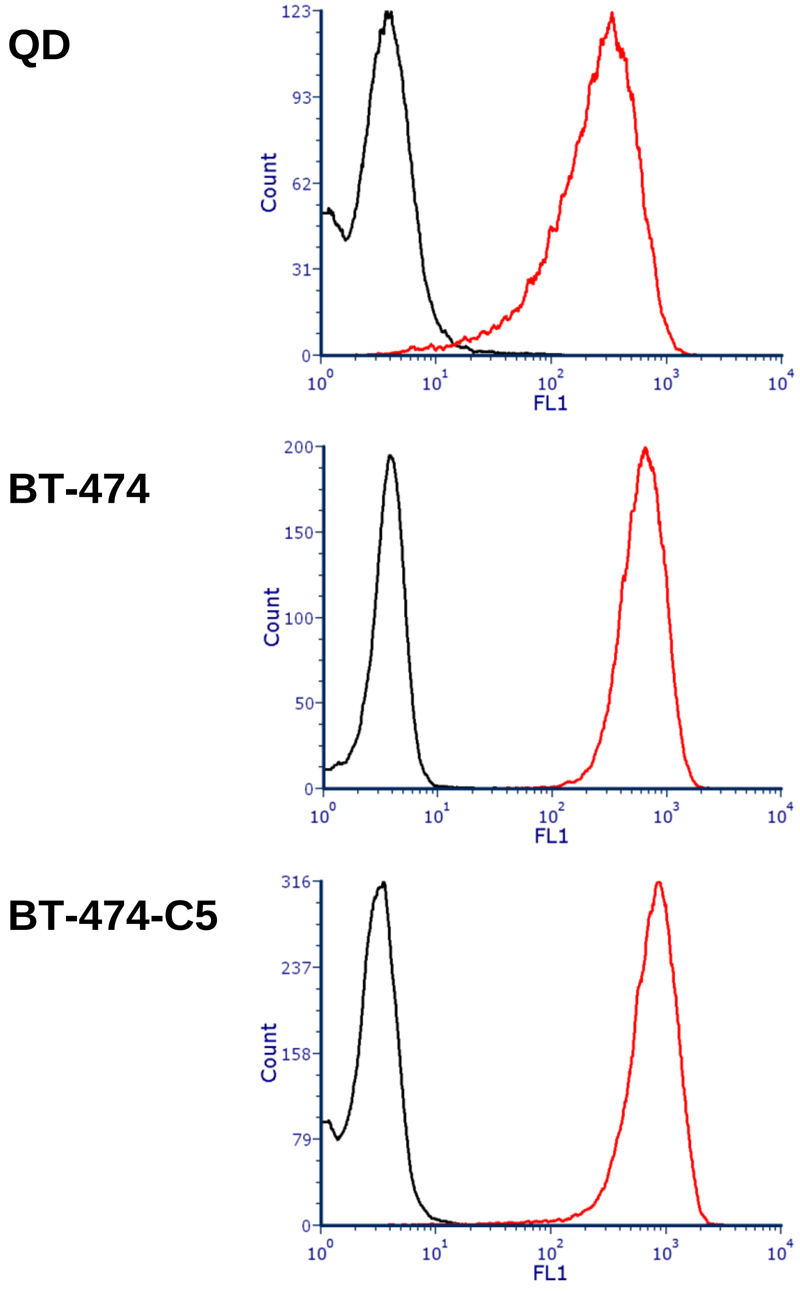

Supplementary Figure S1. HER-2 expression of human HER-2 transgenic mammary carcinoma cell line QD, of human breast cancer cell line BT-474 and of its trastuzumab-resistant clone C5, as determined by indirect immunofluorescence and flow cytometry. Red profile: anti-HER-2 monoclonal antibody MGR-2 + anti-mouse Ig AF488; black profile: anti-mouse Ig only.

Supplementary Figure S2

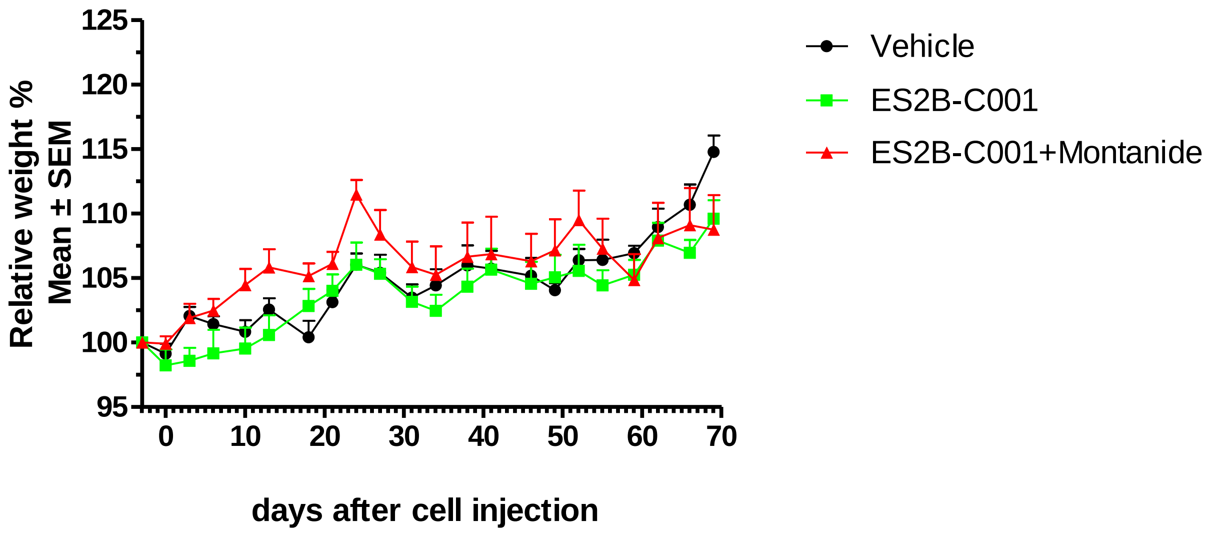

Supplementary Figure S2. Weight curves of FVB mice challenged i.m.f.p. with QD cells and vaccinated with ES2B-C001. *See* Figure 1 for tumor growth in these mice and Figures 3 and 4 for antibody kinetics.
